# Atlanta Society of Medicine

**Published:** 1892-04

**Authors:** C. D. Roy, R. R. Kime

**Affiliations:** Secretary; Reporter


### ATLANTA SOCIETY OF MEDICINE.

MEETING OF MARCH 1ST, 1892.

President W. S. Kendrick in the chair.

DR. INGHRAM reported his experience with apomorphia. A young lady 25 or 30 years old, drunk, hysterical and hard to control. Gave her apomorphia, 1-10 gr. hypodermatically. Patient vomited freely, relaxed and rested quietly. A well-known business man took a large dose of bromidia, and became violently insane; required three men to control him; tried to kill himself and others. Gave 1-10 gr. of apomorphia. He soon vomited, bowels moved, relaxed, and relieved mental condition. Slept well remainder of night. I have used apomorphia in one case of epilepsy—woman aged 40, cataleptic. It acted well. Have used it in bilious colic. An Irishman had a very severe attack; gave 1-10 gr., which caused him to vomit about one gallon of partially digested food, with marked relief. Used it in one case of eclampsia without effect. Patient died about one week afterward. In alcoholism it vomits patient freely. Have used it also in whooping cough to relax spasmodic attacks.

DR. DUNCAN—At 4 a. m. this morning was called to a case of labor. Found patient up; waters had escaped, os dilatable but not dilated fully. I soon dilated os, but head would not engage in pelvis. It was a natural vertex presentation, head not unusually large. Passing finger up posteriorly, found a body just back of head, which was slightly movable between pains. Waited 2 1-2 hours, then applied forceps and delivered. Upon further examination, found another child, feet presenting, which was soon delivered.

He also reported a case of meningitis, 16 years old, colored male. It was reported to be a case of pneumonia. Called last Saturday, and found a case of meningitis, with cervical glands enlarged, opisthotonous and a syphilitic element to deal with.

Gave usual treatment for meningitis. Patient died in three days.

DR. WILLIS WESTMORELAND reported two cases of tracheotomy for foreign bodies. One, a girl of 8 years, swallowed a piece of knitting needle bent in shape of a hair pin. Cut down and found it without much difficulty. The second swallowed a cocklebur four days previously. Performed tracheotomy. Could not find the foreign body; cut two more rings of trachea; kept wound open and the cocklebur came out four days after operation. Left wound open, but did not use tracheotomy tubes; simply attached silk thread on each side of wound in place of tube, which acts better, allows better opportunity for escape of foreign body and no tube left in to irritate parts. Both cases recovered.

DR. KIME reported a case as follows: Was called February 9th to attend Mrs. A., age 23 years, in first confinement. Arrived at 6 a. m. to find child born, cord severed. Removed placenta without any difficulty, which looked to be complete. She was taken at 2 a. m.; in labor four hours. There was a slight laceration of perineum, but did not deem it sufficient to demand sutures. On second day after labor, noticed a slight elevation of pulse, which increased gradually five to eight beats a day until the evening of the sixth day, when she had the first elevation of temperature, 99 3-5 deg. Pulse reached 116 next day, with a temperature of 100 deg. F. On evening of the sixth day I examined patient and found uterus anteverted and os contracted, both interfering with drainage and entering uterine cavity. The evening being dark, I simply dilated cervix and irrigated uterine cavity with $HgCl_2$, 1 to 2,000. Next morning I again dilated cervix, disinfected uterine cavity, and curetted, removing some small placental fragments with free hemorrhage. Again irrigated, following with tincture iodine injected into uterine cavity. Irrigated uterine cavity that evening and twice next day, introducing suppositories containing iodoform, 10 gr.; boric acid, 15 gr. The temperature reached 100 3-5 deg. p. m., after which it gradually subsided as follows: February 18th, 100 1-5 deg.; 19th, 99 4-5; 20th, 99 deg.; 21st, 98 4-5. The intra-uterine irrigations once a day were then discontinued, keeping up hot water vaginal irrigations twice each day with $HgCl_2$, 1 to 2,000, preceded and followed by plain hot water irrigations. Lochia ceased entirely, patient sitting up all day. On February 27th, the 18th day after confinement, temperature again reached 100 3-5 deg. Found uterus anteverted, internal os firmly contracted. On dilating it, two or three tablespoonsful of bloody, purulent, fœtid discharge escaped. Disinfected with mercuric chloride irrigation, hot water and tincture iodine. The next day temperature about the same. Curetted again and irrigated, removing also some small pieces of structure with curette forceps, which seemed too firm for the uterine structures; entirely so for placental structures. With it, also, by use of curette, some shreds of tissue came away resembling partially decomposed uterine mucous membrane. Uterus about 3 3-4 or 4 inches in depth after curetting. Applied pure carbolic acid thoroughly to interior of uterus. Tried to pack uterine cavity with iodoform gauze, but parts were so excessively tender, position of the uterus and contracted os such as to forbid. Next day temperature 99 2-5 deg., highest pulse rate 92. Since then have irrigated uterine cavity twice a day and applied camphor-phenique thoroughly to interior of uterus. Last night, the 21st day after confinement, temperature 99 1-5, pulse, 90.

REMARKS.—For the first eight or ten days after labor, except first, I gave her ergot, then discontinued it, until three days ago I commenced it again. She has taken tonics, quinine and good nourishment. Appetite good, food digests well; bowels require a slight laxative—salines usually given. Feels well and wants to get up all the time. No chills, tongue slightly furred, no pain. No secondary deposits from inflammation, so far as I was able to detect, unless a small place at base of left broad ligament. Every time cervix is dilated, or anything done in uterine cavity, have considerable bleeding. From history of case, quick labor, want of contraction of body of uterus, with contracted cervix, free bleeding on dilatation or entering cavity of uterus, and character of material removed by curette and curette forceps, I am inclined to believe there is a local hyperplasia of uterine muscular tissue or small fibroma of uterine wall. She has always suffered severely at her menstrual periods, wasting more at flows for the last year or two. First flow at 14 years, then missed two years. Tried various physicians to no good, then used copperas, one teaspoonful, brown sugar, one teaspoonful, to one pint of water, taking one tablespoonful three times a day, which established flow. One can easily understand how a deposit of this character would interfere with contractions of the body of the uterus, while the circular fibres of the internal os would be firmly contracted, preventing drainage. It has been said "a septic uterus is a relaxed uterus and patulous os." In this case we have a contracted os of a uterus containing putrid material, and more properly classed as putrid infection.

C. D. ROY, M. D., Secretary.

R. R. KIME, M. D., Reporter.

